# A transdisciplinary dual degree curriculum yields novel and successful learning outcomes: early lessons from training physicianeers

**DOI:** 10.3389/fmed.2025.1520976

**Published:** 2025-02-28

**Authors:** Gregg B. Wells, Douglas A. Baxter, Leslie J. Day, Timothy B. Boone, Michael R. Moreno, Jeremy L. Gibson, Thomas V. Peterson, Margarita Martinez-Moczygemba, Ericka P. Greene, Nicholas Sears, Michael A. Paolini, Roderic I. Pettigrew

**Affiliations:** ^1^School of Engineering Medicine, Texas A&M University, Houston, TX, United States; ^2^Department of Cell Biology and Genetics, College of Medicine, Texas A&M University, College Station, TX, United States; ^3^Houston Methodist Hospital, Houston, TX, United States; ^4^Department of Mechanical Engineering, College of Engineering, Texas A&M University, College Station, TX, United States; ^5^Department of Translational Medical Sciences, Texas A&M University, Houston, TX, United States

**Keywords:** multidisciplinary, interdisciplinary, transdisciplinary, innovation, engineering medicine, convergence

## Abstract

The evolving needs in healthcare education and delivery have led to diverse MD-based dual degree programs offering trainees broader experiences and credential-based credibility after graduation. Medical schools typically implement multidisciplinary or interdisciplinary dual degree training with designs that separate the contributing disciplines chronologically and experientially. As a result, these designs fail to maximize the cohesive learning environment and outcomes possible with a transdisciplinary dual degree design, which integrates the contributing disciplines chronologically, experientially, and conceptually. Though rare, transdisciplinary dual degrees promise transformative educational outcomes and discipline convergence by dissolving traditional discipline boundaries and fostering a new learning environment and professional identity. Therefore, we hypothesize that a transdisciplinary dual degree curriculum yields novel—and potentially better—learning outcomes. ENMED, a transdisciplinary dual degree program collaboratively developed, sponsored, and implemented by Texas A&M University and Houston Methodist Hospital, is testing this hypothesis by training “physicianeers.” This new type of healthcare professional trains simultaneously for the MD and Master of Engineering degrees, thereby integrating medical and engineering expertise to advance health system innovations. Supporting the hypothesis, ENMED’s early experiences suggest its transdisciplinary dual-degree model leads physicianeer trainees to novel perspectives with the potential to transform healthcare systemically.

## 1 Introduction

The US healthcare system is one of the largest in the world with a workforce of highly trained specialists and a robust research sector that historically is associated with improvements in outcomes for significant portions of society. However, the system is challenged with providing access across diverse populations and high costs associated with the delivery of care and treatment, especially as advances in research and innovation have often outpaced funding resources and coverage. The complexity of the system provides the context for both its strengths and weaknesses. It encompasses multiple components that operate independently and, at times, collaboratively, such as federal and state-supported or regulated programs, private sector stakeholders, and ambulatory and hospital-based facilities (1). Additionally, the pipeline of education and training is challenged with producing future healthcare professionals who can solve this system’s complex problems.

A common approach for solving complex problems in healthcare is a multidisciplinary approach, where people from multiple disciplines work in parallel (2–4). Originating in the academic environment, disciplines “are kinds of collectivities that include a large proportion of persons holding degrees with the same differentiating specialization name, which are organized in part into degree-granting units that in part give degree-granting positions and powers to persons holding these degrees” (5). Examples of contributing disciplines are health professions (e.g., medicine, nursing, pharmacy), basic sciences, engineering, social work, architecture, economics, and business. Although people from different disciplines want to develop solutions to the same problem, their solutions are often uncoordinated and unintegrated because they bring different technical languages, solution methods, incentives, and goals and think within single discipline boundaries and conceptual silos (6).

A second approach to complex problem-solving in healthcare is interdisciplinarity with its cooperative analysis and synthesis of problem-solving approaches among disciplines. Experts, each representing a different discipline, reach a consensus across disciplines on a coordinated and harmonious solution. For example, a tumor board decides on a patient’s therapy (7, 8), reflecting diverse expertise and perspectives across discipline boundaries (6). Interdisciplinary problem-solving brings together extensively trained individuals who contribute their diverse discipline-based problem-solving methods (9).

A third and more upstream approach to solving complex healthcare problems is MD-based joint degree or combined dual degree training (10, 11). In principle, a person trained multidisciplinarily or interdisciplinarily in two disciplines will think innovatively across the disciplines’ boundaries with the disciplines’ competencies and knowledge when encountering healthcare problems after training. Another potential benefit is an additional degree-based credential after graduation supporting residency matching (12) or leadership aspirations (13, 14). Dual degree examples combining MD degrees are MD/PhD (15, 16), MD/JD (17), MD/MBA (14), MD/MPH (18, 19), and MD/MS degrees. About 7% of MD graduates in 2024 also earned a graduate (master’s or doctorate) degree (20).

However, the extent of the trainee’s integrated and coordinated learning between the disciplines depends on the dual degree curriculum design and the trainee’s initiative. If integrated and coordinated learning is negligible, the trainee must independently develop thinking skills that merge disciplines and cross discipline boundaries. In contrast, integrated learning can be substantial within a dual degree curriculum when the learning experiences of the disciplines are closely coordinated, instructors cross discipline boundaries, and the learning environment fosters integration. If integrated learning across disciplines is extensive, the dual degree training can be described as transdisciplinary (21), a term describing the dissolving of discipline-based boundaries that generates “fundamentally new conceptual frameworks, hypotheses, theories, models, and methodological applications that transcend their disciplinary origins, with the aim of accelerating innovation and advances in scientific knowledge” (22). Transdisciplinary dual degree training seems ideal for solving complex healthcare problems through convergence (23–27). Two questions accompany this optimism: Under what conditions does such training succeed, and what can it achieve?

## 2 Hypothesis

ENMED (a portmanteau of “engineering” and “medicine”) (28), was founded in 2016 as a collaboration between Texas A&M University and its clinical partner Houston Methodist Hospital in the Texas Medical Center. It was envisioned and developed as a transdisciplinary dual MD/Master of Engineering (MEng) degree program for all its students, with its inaugural class matriculating in 2019. It originated as a parallel track for the MD degree in the Texas A&M University College of Medicine and as the MEng degree, focusing on engineering innovation in medicine (29), in the College of Engineering. ENMED is based on the hypothesis that its chronologically, experientially, and conceptually integrated dual degree curriculum yields novel and successful learning outcomes that positively impact health care. The transdisciplinary combination of engineering and medicine leads to innovative graduates who identify and solve simple to complex healthcare problems. ENMED gives these graduates the name “physicianeers.”

This hypothesis mixes what appear to be two opposing concepts—"transdisciplinary” and “dual degree.” Transdisciplinary training merges and dissolves discipline boundaries. By extension, no existing discipline labels should suitably describe a transdisciplinary degree. On the other hand, training leading to MD and MEng degrees seems to need discipline-identified learning experiences that maintain discipline boundaries and qualify a graduate for both degrees. Despite the model’s name, we find that a transdisciplinary dual degree model fits ENMED’s mission, vision, and discipline-merging realities. This article describes ENMED’s early experiences developing, delivering, and refining a transdisciplinary dual degree curriculum for physicianeering. These experiences might equip other nascent engineering medicine programs that include the MD degree to choose among the options for integrating engineering and medicine.

## 3 Transdisciplinary dual degree curriculum design—chronological and experiential integration culminating in a conceptual merger of disciplines

To explore the hypothesis more deeply, we first describe multidisciplinary and interdisciplinary dual degree options in terms of chronological, experiential, and conceptual integration and then compare them to a transdisciplinary dual degree model. As the origin for our integration reference frame, an education-focused description of disciplinary is, “Concepts and skills are approached separately, allowing students to engage and be assessed on a singular discipline, with minimal integration” (30).

### 3.1 How multidisciplinary and interdisciplinary dual degrees integrate disciplines chronologically, experientially, and conceptually

As a definition, “Multidisciplinarity draws on knowledge from different disciplines but stays within their boundaries” (6). Additionally, an education-focused description of multidisciplinary is, “A common theme or approach is used to allow students to connect concepts and skills learned separately in each discipline. Multiple disciplines are incorporated but they are not *integrated*” (30).

A practical, integration-focused description is that a multidisciplinary dual degree program separates discipline-based training chronologically and experientially. For example, the MD student takes a leave of absence from MD training that occurs with a large cohort of MD-only students—often immediately before or immediately after clerkships—while exclusively pursuing the second degree [called an intercalated second degree (31) outside the United States]. MD/PhD or MD/MBA training usually has this design. Although the need for clinical training continuity during the PhD training years is a recognized need and challenge (32), such clinical training continuity is often implemented with little experiential or conceptual integration with research training. As an advantage, this design is easier for an institution to administer and for instructors to implement because cross-disciplinary interactions are minimized. A trainee independently experiences two discipline-focused learning environments, a design that favors learning and skill acquisition in each discipline. This leave of absence design, however, has implications for dual degree trainees. The dual degree trainee starts MD training with the learning environment and relationships of a matriculating cohort of MD-only trainees. After a leave of absence for the second degree, the dual degree trainee completes and graduates from MD training with the learning environment and relationships of a different cohort of MD-only trainees. For example, serially completing an MD degree and Master of Science degree in an engineering discipline (e.g., biomedical engineering) often requires 5 years in contrast to the usual 4 years for only MD-degree training (33). Moreover, the dual degree trainee typically is not training for either degree with an entire class of like-minded, dual degree trainees, potentially diluting synergies that could arise among like-minded trainees. Additionally, if mentors are not experienced in dual degree thinking, a trainee might acquire less experience with applying dual degree training to complex problems benefitting from interdisciplinary or transdisciplinary solutions compared to the experience acquired from training with mentors who emphasize dual degree thinking. To partially improve discipline integration, a multidisciplinary dual degree design can concurrently deliver discipline-based courses to chronologically merge the disciplines in a student’s schedule. For example, courses leading to an MS degree in an engineering discipline could be scheduled as electives during the pre-clerkship or post-clerkship phases of MD training. Nevertheless, this chronologically merged design neglects to experientially and conceptually integrate the two disciplines.

As a definition, “Interdisciplinarity analyzes, synthesizes and harmonizes links between disciplines into a coordinated and coherent whole” (6). Additionally, an education-focused description of interdisciplinary is, “Students learn concepts and skills from two or more disciplines that are tightly linked so as to deepen knowledge and skills. Educators and learners collaborate to identify a concept involving multiple disciplines in an integrated way that makes the concept authentic and real-world” (30).

A practical, integration-focused description is that an interdisciplinary dual degree program integrates the two disciplines chronologically and experientially in courses and practicums. Faculty members represent their disciplines and coordinate their teaching across disciplines while retaining their discipline-centric professional identities. For example, team-teaching and co-teaching are approaches to chronologically and experientially integrating interdisciplinary education (34–36). Successful implementation necessitates interdisciplinary planning and faculty development to cultivate interdisciplinary mindsets among faculty members.

A variation on an interdisciplinary dual degree is an interdisciplinary approach to an MD-only degree. For example, the Carle Illinois College of Medicine includes an engineering and transformative innovation focus in its MD degree curriculum (37, 38). Its courses are designed and co-directed by teams including a basic scientist, a clinician, and an engineer (39). The University of Texas at Austin Dell Medical School includes leadership and transformative healthcare problem-solving in its MD-degree curriculum, which includes opportunities to earn a master’s degree inside the four-year MD-degree curriculum (40–42). The Vanderbilt University School of Medicine’s Medical Innovators Development Program includes emphases in engineering, translational development, and innovation in its MD-degree curriculum (43). The University of Massachusetts T.H. Chan School of Medicine has implemented an MD curriculum with an Entrepreneurship, Biomedical Innovation, and Design pathway (44).

### 3.2 How transdisciplinary dual degrees integrate chronologically, experientially, and conceptually

As a definition, “Transdisciplinarity integrates the natural, social and health sciences in a humanities context, and transcends their traditional boundaries” (6).

An education-focused and distinguishing description of transdisciplinary is:

"Learners identify complex problems and work together to create a shared conceptual framework and draw together theories, concepts, and practices that transcend individual disciplinary boundaries. Focus is on broad, real-world constructs drawn from an increasingly interconnected world, societal relevance, and student interest. Transdisciplinary (including applied interdisciplinary approaches) is distinct from multi-or inter-disciplinary in that subjects are blended in a transformative manner that provides important gateways for student-centric, student-defined problems or topics that lead to authentic and meaningful learning experiences and student-driven innovations" (30).

A practical, integration-focused description is that the two degree-awarding disciplines of a transdisciplinary dual degree program provide essential foundations for the increasingly integrated continuum of multidisciplinary, interdisciplinary, and transdisciplinary education (30). Transdisciplinary dual degree training builds on these foundations but is not limited by them. Instead of a leave of absence bridging two disciplines, this program tightly integrates the two disciplines chronologically, experientially, and conceptually. In transdisciplinary learning’s purest form, teaching and learning dissolve discipline boundaries and focus on solving real-world problems with skills and approaches drawn from both disciplines and novel skills and approaches that transcend the limits of both disciplines (45).

Another manifestation of transdisciplinary learning is nearly instantaneous transitions in thinking between and beyond the two disciplines. For example, learners and instructors seamlessly move between and beyond disciplines during learning, innovation, and problem-solving. Transdisciplinary learning places unique demands on faculty members and faculty development. They must learn to think and teach transdisciplinarily, even if they start with only single-discipline expertise. Unique demands also are placed on the learners. They must become equipped with the two discipline-based foundations while also learning in the multidisciplinary, interdisciplinary, and transdisciplinary continuum.

Determining measures of a program’s success can be challenging because of the pioneering nature of a transdisciplinary dual degree program. The measures, in part, come from the disciplines’ traditional measures. However, they must also go beyond the disciplines and into the convergence that addresses healthcare problems.

## 4 ENMED’s transdisciplinary dual degree: training physicianeers

Among the dual degree design options, ENMED is following a transdisciplinary dual degree design for all its students because of its mission and vision to train physicianeers, a new type of healthcare professional, through the merging of engineering and medicine (46). Its mission is “to develop a new healthcare professional, trained to be an exceptional physician who is also equipped to invent practical solutions to healthcare problems through the convergence of engineering and medicine. Such innovators will be Physicianeers.” Its vision is that, “ENMED graduates will uniquely help to transform healthcare as Physicianeers. This will be achieved through convergence born innovations that improve the understanding and treatment of disease.” To facilitate students’ completion of this dual degree training within 4 years, ENMED requires its students to have earned a baccalaureate degree in engineering or computer science or have completed mathematics and engineering leveling courses (47). Thus, students enter ENMED with a foundation in engineering-oriented thinking. They build on that discipline-centric foundation to earn the MD and MEng degrees through increasingly transdisciplinary experiences across the curriculum timeline (48). In addition to program objectives for MD and MEng degrees, ENMED also has these four physicianeering objectives:

- Integrate basic medical and clinical sciences with engineering concepts.
- Critically analyze current practices in order to identify opportunities to develop innovative solutions to problems in medicine and health care.
- Demonstrate an understanding of key concepts needed for the implementation of medical technologies in health care.
- Demonstrate the ability to form teams with relevant transdisciplinary expertise and work collaboratively to develop and implement solutions to real-world medical problems.

Faculty, students, staff, and the learning environment implement ENMED’s curriculum and achieve ENMED’s physicianeering objectives. Courses, including clinical training, have discipline-specific aspects that contribute expertise and course credits to either degree, but engineers, basic scientists, and clinicians collaborate with course planning and execution throughout the four-year curriculum [see (48) for more information about ENMED’s curriculum model]. The curriculum initially employs multidisciplinary and interdisciplinary approaches, including the Stanford Biodesign process (49), to introduce students to engineering medicine. As students encounter increasingly complex healthcare problems, faculty members encourage students to think increasingly across disciplines through curricular activities designed for applying integrated knowledge across basic and clinical science and engineering (e.g., team-based learning and self-directed learning).

Students’ shared experiences from engineering backgrounds contribute to a learning environment committed to learning physicianeering and forming a professional identity as a physicianeer. The faculty members’ mindset also is essential to this learning environment. Although they come from basic medical science, engineering, and clinical disciplines, they acquire an increasing transdisciplinary mindset through their interactions across disciplines and with students. Faculty members’ transdisciplinary development trajectory shares features with, but continues beyond, interdisciplinarity as faculty members become increasingly adept at thinking across discipline boundaries and like physicianeer instructors. Staff members’ transdisciplinary thinking and practices support ENMED’s dual degree and transdisciplinary goals. Overall, the learning environment committed to physicianeering distinguishes ENMED from most other dual degree settings in which relatively few students share dual degree goals in an overall learning environment dedicated to only one degree (48).

Two activities principally are intended to put transdisciplinary learning into practice. Three 4-week-long Innovation Immersion Experiences (IIE) and the five semesters of Engineering Analysis of Clinical Processes (EACP) during clerkships and the transition to residency phase provide environments for students to devise engineering solutions to clinical problems. The projects are overseen by engineering faculty and clinicians to ensure relevance and fulfill Stanford Biodesign’s need statement (49–51).

Students enrich their learning by interacting with biomedical inventors, entrepreneurs, and biodesigners (49, 52). They explore and realize their creative ideas in the Engineering Innovation Center (EIC; an engineering makerspace) and the Biological Engineering Maker Space, a cellular-and molecular-based counterpart to the EIC, while learning basic science, clinical, and innovation-engineering curricular content. Their transformational mindset is expected to help students develop skills for addressing real-world healthcare problems.

## 5 ENMED’s early outcomes—successes and challenges

### 5.1 Successes

As tracked by Texas A&M University and Houston Methodist Hospital, currently available measures of success for the ENMED program include several established outcome measures used by medical education and biodesign engineering programs. ENMED’s outcomes, with its almost 200 matriculated students and its two graduated classes of 2023 (22 members) and 2024 (33 members), thus far support the hypothesis. The mean USMLE Step 2 scores of the two graduated classes exceeded the national average. Some students are highly clinically oriented, while others are highly oriented to translational innovation and commercialization. All graduating students were matched for post-graduate residency training.

ENMED faculty and students published more than 60 articles and presented at more than 60 conferences during 2023 and early 2024. ENMED students produced six intellectual property disclosures in 2022 and 16 in 2023. They have filed seven provisional patents and five Patent Cooperation Treaty patents. A student-led company was formed to commercialize an invention.

### 5.2 Challenges

Many of ENMED’s challenges arise from its unprecedented goal of training physicianeers in 4 years. Students must have an engineering or computer science baccalaureate degree or equivalent. This prerequisite helps reduce cognitive overload and facilitates earning the MEng and MD degrees concurrently but constrains the pool of eligible applicants. Faculty must embrace a transdisciplinary mindset, which is demanding given the acquired traditional expertise in a distinct basic science, engineering, or clinical discipline. Faculty members must learn how to teach physicianeering without an established template. No existing curriculum, faculty development, and career paradigms exactly fit this goal, and blazing this new path with its dual MD/MEng degrees completed in 4 years requires innovative thinking and experimentation. For comparison, physician-scientists have recognized training and independent career paths, often in academic medicine (53). In contrast, training for physicianeers continues to unfold at ENMED. Moreover, career paths of ENMED graduates have yet to be fully realized because physicianeers are a new category of healthcare professionals. An emerging alternative to ENMED’s dual degree is an MD-degree-only approach to training for healthcare innovation as a “physician-innovator.” Many US medical schools are developing this approach (37, 40, 44, 54–59). Accumulating experience will help identify contexts and career goals that favor dual degree “physicianeer” versus single-degree “physician-innovator” training.

## 6 Limitations of ENMED’s hypothesis testing

Support for the hypothesis from our ENMED experience has limitations. First, ENMED is only one implementation of an MD-based transdisciplinary dual degree program specializing in MD and MEng degrees. Transdisciplinary dual degree experiences from other institutions and discipline combinations with an MD degree would more broadly explore the hypothesis and its scalability. Second, qualitative and quantitative research data from students, faculty, and graduates will more deeply explore the hypothesis and provide a better understanding of how ENMED works and career paths of ENMED’s graduates. Third, ENMED’s experience is relatively short term. Long-term outcomes from ENMED’s relationships with healthcare ecosystem partners will provide important tests for the hypothesis. These partners include patients and their families, graduate and subspecialty medical education, academic medical centers, biomedical innovation centers (60, 61), and biotechnology.

## 7 Conclusions, questions, and future work

Our ENMED experiences highlight three important elements of transdisciplinary dual degree programs. First, students need tailored preparation for transdisciplinary dual degree training beyond medical school prerequisites. ENMED students have a baccalaureate degree in engineering or computer science, equipping them for master’s degree-level training in engineering when they start ENMED. Students must be eager for the three professional identities they will acquire—physician, engineering professional, and physicianeer. Second, faculty must be eager to transcend their discipline’s familiar boundaries, be willing to create and deliver educational materials transdisciplinarily, and be able to develop a transdisciplinary professional identity. Third, the learning environment must be transdisciplinary and encourage students and faculty to be, think, and work unconstrained by discipline foundations and silos.

ENMED’s early experiences have raised further questions about transdisciplinary dual degree programs. First, what paradigms can guide transdisciplinary dual degree programs’ creation and evolution? Interdisciplinarity is an initial concept but can only be a starting paradigm for a practical design. Second, on what timeline might a transdisciplinary dual degree evolve into an academic and professional discipline distinct from the two foundational disciplines? Third, is a transdisciplinary dual degree program realizable in the context of discipline-based dual degrees? ENMED’s early experiences answer this question in the affirmative. More experience is needed before judging whether an interdisciplinary or transdisciplinary single-degree design is preferred to a dual degree transdisciplinary design. Judgment will probably depend on a specific program’s goals, resources, and context.

Transdisciplinarity intends to solve a range of complex problems, which are abundant in healthcare. Supporting the hypothesis, ENMED’s experiences and outcomes show its transdisciplinary dual degree design has a promising beginning for contributing effective solutions.

## Data Availability

The original contributions presented in the study are included in the article/supplementary material, further inquiries can be directed to the corresponding author.
